# Effects of Β-blocker Administration on Cardiac Function: A Coronary Computed Tomography Angiography Study

**DOI:** 10.2174/1573405618666220518104929

**Published:** 2022-11-07

**Authors:** Reiji Kokubo, Masaharu Hirano, Yu Tajima, Daisuke Yunaiyama, Kazuhiro Saito

**Affiliations:** 1 Department of Radiology, Tokyo Medical University, Tokyo, Japan;; 2 Department of Cardiology, Tokyo Medical University, Tokyo, Japan

**Keywords:** β-blocker, Echocardiography, Coronary computed tomography angiography, Ejection fraction, Heart rate, Heart failure

## Abstract

***Background*:** β-blockers are widely used for lowering heart rate (HR) during coronary computed tomography angiography (CCTA); however, they should be used with caution for patients with heart failure as they may have a negative inotropic effect.

***Objective*:** To clarify the effects of β-blockers (oral and intravenous injection) on cardiac function using CCTA.

***Methods*:** A total of 244 patients (men: women = 166: 78; mean age, 64.4 years old) suspected of having ischemic cardiac disease and had undergone echocardiography within 3 months before and after CCTA were included in the study. Systematic errors in ejection fraction (EF) were corrected by calculating ΔEF from the EF difference between echocardiography and CCTA in patients not using β-blockers. Univariate and multivariate analyses were performed for factors affecting ∆EF. In addition, HR between, before, and during CCTA were compared by Wilcoxon’s test.

***Results*:** Temporary oral or intravenous administration of β-blockers at the CCTA had no significant effects on EF (p = 0.70), whereas HR was significantly decreased (p < 0.001). However, regular administration of β-blockers increases the EF on CCTA.

***Conclusion*:** The administration of β-blockers immediately before CCTA affects HR but not EF. Premedication with β-blockers can be safely used for patients who undergo CCTA, and CCTA is useful for EF evaluation, independent of the use of β-blockers.

## 1 INTRODUCTION

Ischemic heart disease is usually diagnosed using echocardiography, the exercise stress test, stress myocardial scintigraphy and coronary angiography. The recent rapid development of diagnostic imaging techniques has enabled minimally invasive visualization of coronary arteries [1, 2]. The use of multidetector-row computed tomography has become widely accepted owing to its high spatial resolution and high throughput [3]. Coronary computed tomography angiography (CCTA) is widely used for screening for ischemic heart disease, the evaluation of complex ischemic lesions, including chronic total occlusion and multiple branch lesions, evaluation before ablation treatment, percutaneous coronary intervention (PCI), and coronary artery bypass grafting (CABG), and follow-up after PCI and CABG [4].

Imaging of the heart is affected by motion artifacts caused by the heartbeat and vertical movement of the diaphragm during respiration. CCTA is therefore performed under respiratory arrest and in synchronization with the heartbeat to reduce these artifacts. However, there is a limitation of the gantry speed during CT, which determines the temporal resolution of CT. The main method used to overcome this problem is heart rate (HR) suppression with β-blockers, where usability has been well established in the literature [5, 6]. This increases the relative temporal resolution [7], and studies have shown that controlling the HR with β-blockers to 60 beats per minute (bpm) or less improves image quality [7, 8]. The method is recommended in the Society of Cardiovascular Computed Tomography guidelines [7].

β-blockers used to induce bradycardia during CCTA imaging are widely administered to treat hypertension and ischemic heart disease. Together with angiotensin-converting enzyme inhibitor (ACE-I) / angiotensin II receptor blocker (ARB) and diuretics, β-blockers play a central role in treating heart failure [9]. On the other hand, β-blockers may have a negative cardiac inotropic effect that results in cardiac dysfunction [10], and they were initially used with caution in heart failure patients. Theoretically, the administration of β-blockers to such patients at the time of CCTA should be considered carefully; however, their safety has been reported without verifying their effects on cardiac function during CCTA [5, 6]. To the best of our knowledge, there is no report that evaluates the effect of β-blocker administration in CCTA patients. Therefore, this study aimed to clarify the effects of β-blockers on cardiac function during CCTA.

## 2 MATERIALS AND METHODS

### 2.1 Declare Section

This retrospective single-center study was conducted in accordance with the Declaration of Helsinki (as revised in 2013). The study was approved by the institutional ethics board of Tokyo Medical University (NO.: T2018-0061), and individual consent for this retrospective analysis was waived.

### 2.2 Subjects

Using the radiological reporting system, we selected consecutive subjects suspected of having ischemic cardiac disease between April 2015 and March 2018 and who had undergone echocardiography within 3 months before and after CCTA. Patients were excluded from the study if they had an implanted cardiac pacemaker or defibrillator; lacked any part of patient characteristics; or had atrioventricular block; atrial fibrillation or extrasystoles at the time of CCTA or echocardiography. In addition, patients who were pregnant, lactating, possibly pregnant, or desiring to become pregnant during the study period were excluded.

### 2.3 Echocardiography

**Fig. (1):**
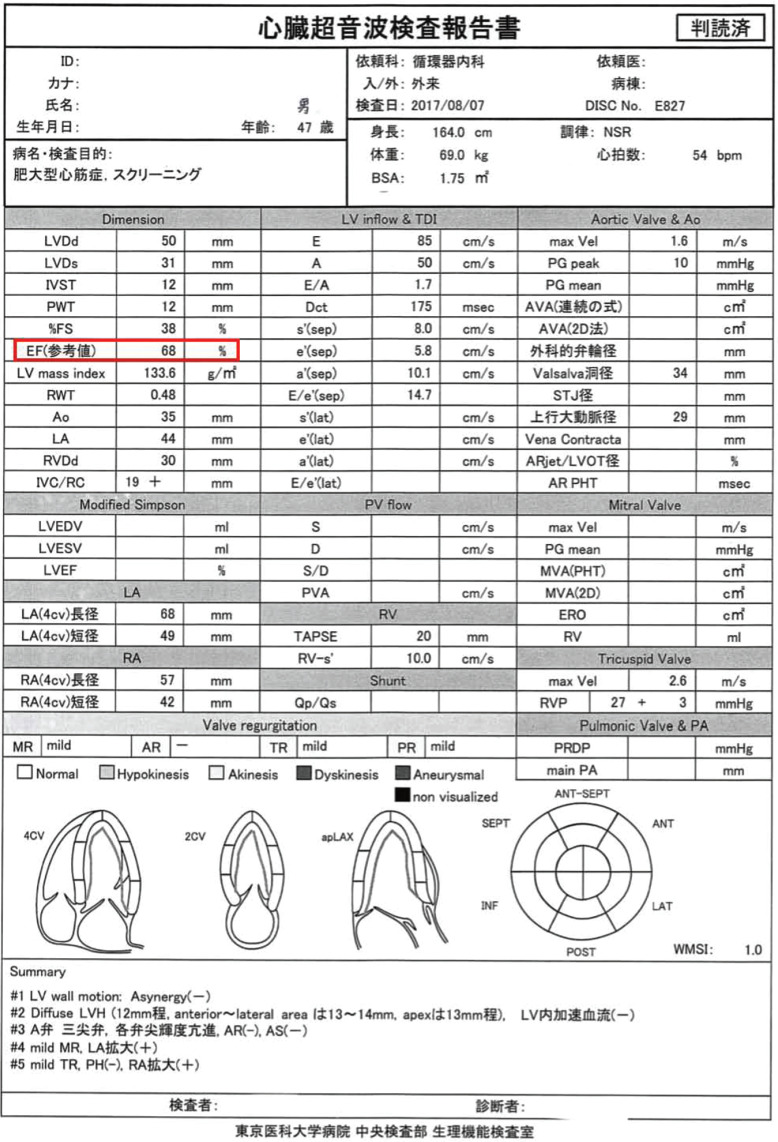
A 47-year-old man was referred to our hospital by a clinician for further examination of heart failure. The patient ordinarily does not take β-blockers. An echocardiogram examination showed 68% of EF.”

Three ultrasound (US) machines were used (Philips EPIQ7, TOSHIBA APLIO XG SSA-790A, and GE Vivid E9) to measure standard references for the analysis of ejection fraction (EF). The mean duration between echocardiography and CCTA was 21.7 days (median: 13 days). All patients were placed in the left lateral decubitus position for the imaging. Images were obtained in standard apical 4-chamber views and 2-chamber views by seven medical technologists who specialized in echocardiography and were blinded to the CCTA data. Left ventricular end-diastolic dimension (DD) and end-systolic dimension (DS) were also measured using M-mode echocardiography. DS, DD, and corrected EF were analyzed (Fig. **1**).

### 2.4 CCTA Protocol

Patients whose HRs exceeded 60 bpm received oral bisoprolol 14 hours before the CCTA and metoprolol 2 hours before the CCTA. Intravenous landiolol was also given if a patient’s heart rate exceeded 55 bpm. CCTA was done with a continuous helical scan, 4 to 7 minutes after the landiolol administration.

The CT equipment used was a single-source 64-MDCT (Lightspeed VCT, GE Medical Systems, Inc.). The rotation speed of the X-ray tube was set to the maximum. Nonionic contrast medium, iopamidol (370 mg/mL), was rapidly injected intravenously at 3 to 4.5 mL/s using a 2-channel injector, followed by 20 to 30 mL saline. The effective doses for the nonenhanced scans and CCTA were estimated from the dose-length product and a conversion coefficient (*k* = 0.014 mSv / (mGy × cm)) for the chest as the investigated anatomical region. Other imaging conditions were as follows: tube voltage, 120 kV; tube current, 500–750 kV; collimation: 64 rows × 0.625 mm; X-ray tube rotation speed: 0.35 second/rotation; helical pitch ≤ 0.24; field of view, 200 mm.

### 2.5 Image Acquisition and Data Analysis

Image reconstruction was performed by the retrospective electrocardiogram (ECG)-gated reconstruction method, with a slice thickness for reconstruction of 0.625 mm.

**Fig. (2):**
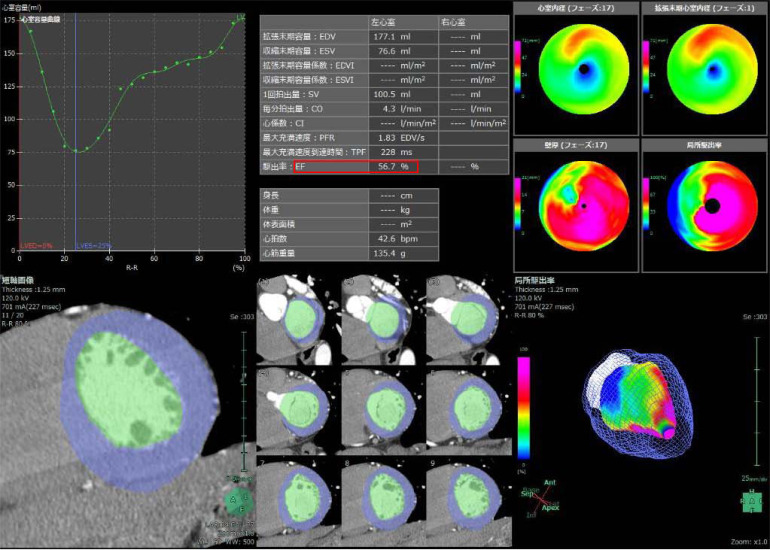
A coronary computed tomography angiography was performed on the same patient referred to in Fig. (**1**), with pre-study β-blockers (bisoprolol and landiolol) The EF was calculated as 56.7%.”

The patient characteristics of sex, age, height, body weight, body mass index, systolic blood pressure, diastolic blood pressure, HR on monitoring, HR on CT, and clinical diagnosis were analyzed. The medication history and use of beta-blockers at the time of CCTA were also analyzed. End-diastolic volume (EDV), end-systolic volume (ESV), EF, myocardial weight, and HR at the time of CCTA were measured by cardiac function analysis using Volume Analyzer SYNAPSE VINCENT (FUJIFILM Medical Co., Ltd., Tokyo, Japan) as the image processor. Image analyses were performed by a cardiologist with 30 years of experience and a radiologist with 5 years of experience, with a consensus reading (Fig. **2**).

**Fig. (3):**
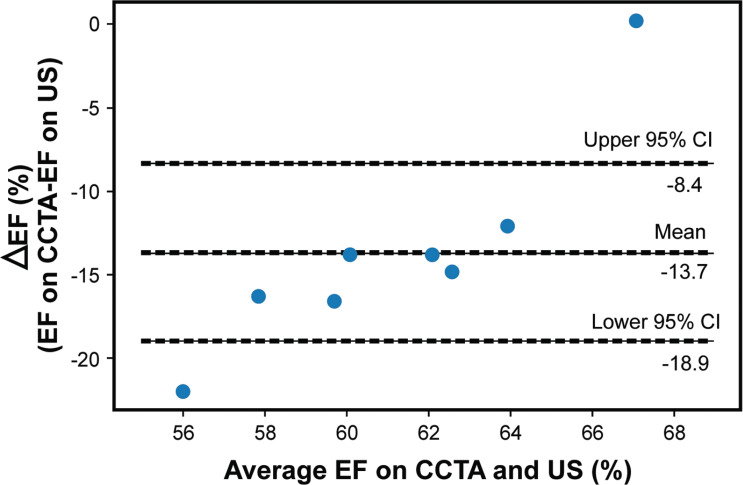
Bland-Altman plot analysis was performed to analyze the presence of systemic errors, and fixed errors were identified. Corrections were made by adding the mean value of ΔEF to the echocardiography data for all patients.

To compare the data from echocardiography and CCTA, data correction was performed based on the same EFs in both modalities by analyzing systemic error using the Bland-Altman plot [11]. The existence of a fixed error was identified, and the correction was made by adding the mean value of ΔEF to the echocardiography data for all patients (Fig. **3**).

Univariate analyses were performed for the patient characteristics, clinical diagnoses, regular drugs, and pre-study β-blockers affecting ΔEF. In addition, multivariate linear regression analyses with ordinary least squares regression were carried out to analyze the effects of possible confounding factors associated with the EF. Multivariate analyses were performed on factors with a *p*-value of less than 0.1 by univariate analysis. Factors with a variance inflation factor (VIF) of greater than 10 were highly multicollinear and were excluded from the multivariate analyses. Patients were divided into two groups, those regularly taking β-blockers and those who were not, and the data were analyzed by the Mann–Whitney U test. We also analyzed the data according to the presence of beta-blocker side effects at the time of examination.

The EF and HR during CCTA in patients who were given oral or intravenous β-blockers were compared with the EF on echocardiogram plus ΔEF and HR on echocardiogram by Wilcoxon’s test, respectively.

All statistical analyses were performed using R software v. 3.5.3 (R Foundation, http://www.r-project.org/). A *p*-value of less than 0.05 was considered statistically significant.

## 3 RESULTS

### 3.1 Study Sample

The study sample consisted of 244 patients, as shown in Table **1**. The clinical diagnosis before CCTA included 62 patients with ECG abnormalities (25.4%), 48 with ischemic heart disease (19.7%), and 10 with cardiomyopathy (4.10%). There were 124 patients classed as “other” (50.8%).

There were 148 patients (60.7%) who were taking medications. Of the patients taking regular medications, 94 took β-blockers (38.5%). A total of 72 patients (48.6%) regularly took more than 2 drugs. Eight patients were not given temporary β‒blockers (bisoprolol fumarate, metoprolol, and landiolol) at the time of CCTA as the HR at CCTA was less than the cut-off line (Table **2**). Pre-study β-blockers were used in 236 (96.7%) cases, of which bisoprolol fumarate was used in 96 (40.7%), metoprolol in 203 (86.0%), and landiolol in 142 (60.2%).

US measurements were DS 31.2 ± 5.38 mm and DD 47.6 ± 4.90 mm. The corrected EF on US was 49.7 ± 8.36%. On CCTA, ESV was 65.8 ± 28.5(mL), EDV was 133 ± 36.3 mL, SV was 66.5 ± 16.1 mL, and CO was 3.52 ± 0.814 L/min). The EF on CT was 51.7 ± 9.61%, and myocardial weight was 107 ± 36.7 g (Table **3**).

### 3.2 Statistical Analysis of Factors Affecting ΔEF

Univariate analysis identified age, landiolol administration, HR on CT, regular use of β-blockers, and ECG abnormalities, as significant factors affecting ∆EF (*p* < 0.05) (Table **4**). HR on CT was excluded from the factors as it was closely correlated with landiolol administration (VIF = 24.0) by multivariate analysis. Multivariate analyses also showed that the regular use of β-blockers and ECG abnormalities were factors affecting EF (Table **5**). Oral and intravenous administration of β-blockers (bisoprolol fumarate, metoprolol, and landiolol) used temporarily at CCTA had no significant effects on ΔEF.

### 3.3 Calculation of ΔEF

The correlation between ΔEF, as EF on CCTA-EF on US (%), and average EF on CCTA and US is shown in Fig. (**3**). The mean value was −13.7 (95% C.I: −8.4–18.9).

### 3.4 The Effect of Temporary Oral or Intravenous Administration of β-blockers on EF and HR

EF on CCTA in patients with temporary oral or intravenous administration of β-blockers showed no significant difference compared with EF on echocardiogram; however, HR showed a significant difference (*p* < 0.001).

### 3.5 The Effects of the Regular Administration of β-blockers

**Fig. (4):**
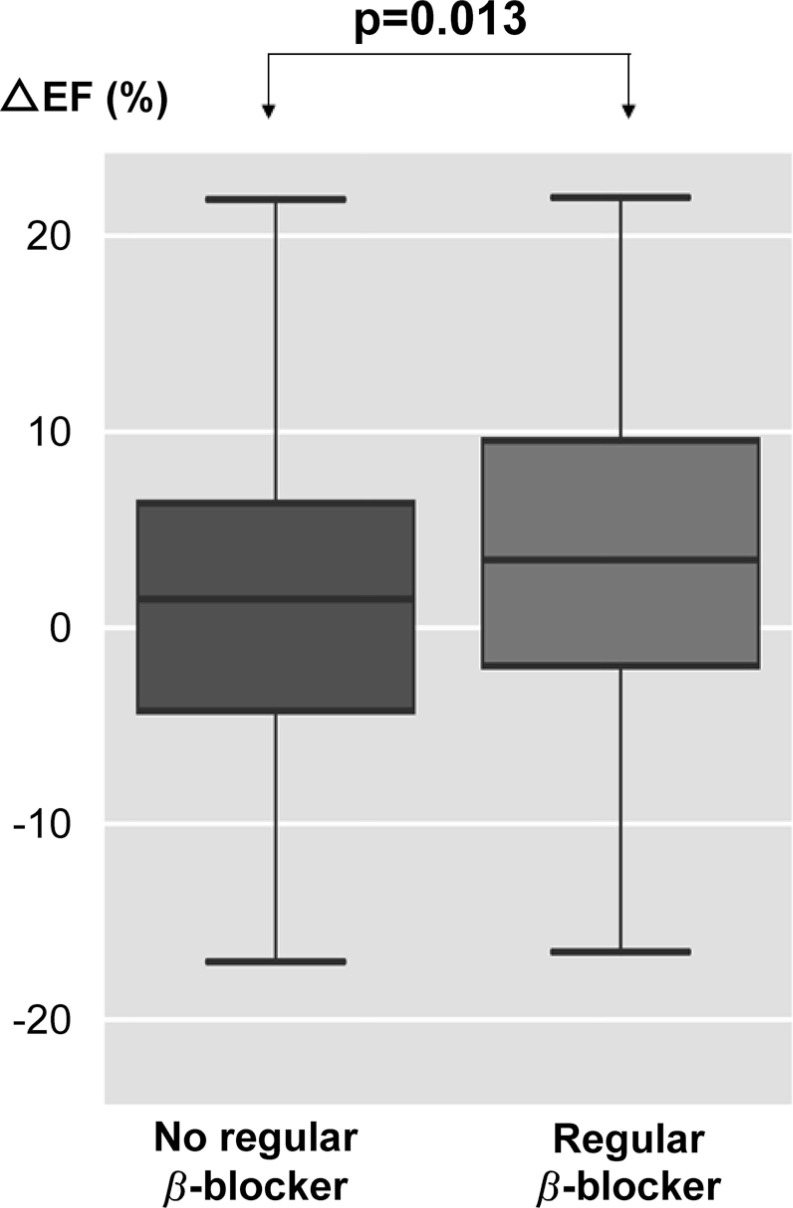
In the group of patients regularly taking β-blockers, ΔEF was significantly increased by 2.7% compared with the group of patients who were not.

The effects of the regular administration of β-blockers on cardiac function are shown in Table **6**. ESV, EDV, and SV during CCTA were significantly higher in the group regularly taking β-blockers. On the other hand, there was no significant difference between DS and DD. A statistically significant difference between the groups was observed in ∆EF. In the group of patients regularly taking β-blockers, ΔEF was 2.7% higher than in the group of patients who were not (*p* = 0.013) (Fig. **4**). On the other hand, the coefficient of ΔEF in the group of patients with abnormal ECGs was 3.1% lower than that of the group of patients without ECG abnormalities (Table **5**). No side effects were noted after the imaging analyses, regardless of the administration of β-blockers or the dose administered.

## 4 DISCUSSION

In this study, the oral and intravenous administration of β-blockers (bisoprolol fumarate, metoprolol, and landiolol) at the time of CCTA had no significant effects on ΔEF. No side effects were noted after CCTA, regardless of the administration of β-blockers or the dose. Therefore, our results suggest it is safe to administer β-blockers as a pretreatment for CCTA. In addition, there have been concerns about a possible excessive decrease in EF in patients routinely taking β-blockers, however, such a decrease was not observed, indicating that β-blockers can be used relatively safely, except for patients who are contraindicated for β-blockers [5, 6].

Left ventricular EF is one of the most important prognostic factors in patients with chronic heart failure [12-14]. Therefore, clinically, the measurement of EF is important. Echocardiography, magnetic resonance imaging (MRI), single-photon emission computed tomography (SPECT), and left ventricular angiography (LVG) can be used to measure EF. Echocardiography is widely used in clinical practice because it is non-invasive and simple to perform. Echocardiography correlates well with the volume determined by left ventricular angiography [15-17]. CT and MRI are less invasive than conventional methods and have higher diagnostic accuracy and reproducibility than echocardiography [18-20]. Recent progress in imaging techniques has enabled less invasive visualization of the coronary arteries in ischemic heart disease patients. In contrast coronary angiography, the number of CCTAs being performed is increasing [3]. CCTA is a useful diagnostic method as it can assess coronary arteries and EF, which predicts a patient’s prognosis objectively, accurately, and less invasively. Whereas HR control is essential for clearly delineating the coronary arteries, and the administration of β-blockers is also important, β-blockers are thought to result in the EF being estimated to be lower than its actual value [21]. However, in our study, the oral administration of β-blockers (bisoprolol fumarate and metoprolol) and intravenous injection of a β-blocker (landiolol) at the time of CCTA had no substantial effects on ΔEF. Therefore, our results demonstrate that CCTA is useful for EF evaluation, independent of the use of β-blockers.

In this study, ∆EF was significantly affected by the regular administration of β-blockers and the presence of ECG abnormalities. The rate of change was −3.1%, even in patients with ECG abnormalities, which was the factor that reduced EF most. Therefore we believe that β-blockers can be administered relatively safely. Risks at the time of CCTA can be determined by checking whether the patient takes β-blockers regularly and whether there are any ECG abnormalities. Therefore, by taking the patient’s medical history, performing a medical examination, and performing an ECG and echocardiography, it is possible to carry out CCTA more safely even when using β-blockers. In our study, patients regularly taking β-blockers showed increased EFs on CCTA. This is probably because a large amount of the contrast agent flows into the myocardium, which is expanded by the β-blocker, resulting in an increase in the preload and a temporary increase in EF compared with US evaluation.

There are some limitations to this study. First, it was retrospective. Prospective or interventional studies will be needed in the future. Secondly, we used different modalities, cardiac ultrasonography and CCTA, to measure cardiac function. It is difficult to measure cardiac function using echocardiography before and after CCTA as the effects of landiolol disappear in up to 30 minutes. In addition, CCTA imaging both with and without the administration of β-blockers in one patient at the same time, is of little clinical significance and is not acceptable from the viewpoint of radiation exposure. Several studies have reported that the analysis of cardiac function by CCTA is comparable to that by cardiac ultrasonography [22]. Wu *et al*. reported that the correlation coefficients of cardiac function measurements by referencing MRI with CCTA and echocardiography showed a high correlation of 0.98 and 0.87, respectively [23]. Although there is a high correlation between the results of CCTA and echocardiography, only comparing CCTA with or without β-blockers is a limitation. Thirdly, we corrected the data using the EF of only a small number of patients who did not receive both oral and intravenous β-blockers at the time of CCTA. In the future, studies should be performed on a larger number of patients who are not taking β-blockers at the time of CCTA.

## CONCLUSION

β-blockers can be safely administered as a premedication for CCTA, and the additional injection of β-blockers just before CCTA does not affect the analysis of cardiac function. However, regular administration of β-blockers may increase the EF on CCTA.

## Figures and Tables

**Table 1:** Patient Characteristics.

Age (years), mean ± SD		64.4 ± 11.8
Sex (male/female), n (%)		166/78 (68.0/32.0)
Height (cm), mean ± SD		163 ± 9.77
Bodyweight (kg), mean ± SD		64.6 ± 14.0
Body mass index (kg/m^2^), mean ± SD		24.0 ± 3.90
Heart rate (beats/min) in the morning, mean ± SD		66.2 ± 9.74
Heart rate (beats/min) at examination, mean ± SD		53.4 ± 7.20
Systolic blood pressure (mmHg), mean ± SD		131.9 ± 19.9
Diastolic blood pressure (mmHg), mean ± SD		77.3 ± 13.0
Clinical diagnosis	ECG abnormalities, n (%)	62 (25.4%)
	Ischemic heart disease, n (%)	48 (19.7%)
	Cardiomyopathy, n (%)	10 (4.10%)
	Others, n (%)	124 (50.8%)

ECG, electrocardiogram; SD, standard deviation.

**Table 2:** Summary of medication use.

**No Regular Drug, n (%)**		**96 (38.7%)**
Regular drug, n (%)	-	152 (61.3%)
	α-blocker, n (%)	19 (7.7%)
	β-blocker, n (%)	96 (38.7%)
	Ca-antagonist, n (%)	70 (28.2%)
	Diuretics, n (%)	18 (7.3%)
	ARB/ACE-I, n (%)	62 (25.0%)
Pre-study β-blocker	-	236 (96.7%)
	Bisoprolol fumarate, n (%)	96 (40.7%)
	Metoprolol, n (%)	203 (86.0%)
	Landiolol, n (%)	142 (60.2%)

ARB, angiotensin II receptor blocker; ACE-I, angiotensin-converting enzyme inhibitor.

**Table 3:** Summary of cardiac function measured by echocardiogram and coronary computed tomography angiography.

**US**	**-**
DS (mm)	31.2 ± 5.38
DD (mm)	47.6 ± 4.90
Corrected EF (%)	49.7 ± 8.36
CCTA	
ESV (mL)	65.8 ± 28.5
EDV (mL)	133 ± 36.3
SV (mL)	66.5 ± 16.1
CO (L/min)	3.52 ± 0.814
EF (%)	51.7 ± 9.61
Myocardial weight (g)	107 ± 36.7

US, ultrasound; CT, computed tomography; DS, end-systolic dimension; DD, end-diastolic dimension; ESV, end-systolic volume; EDV, end-diastolic volume; SV, stroke volume; CO, cardiac output; EF, ejection fraction.

**Table 4:** Univariate analysis for factors affecting ΔEF.

**Variable**		**Adjusted Relative Risk**	**95% CI**		***p*-value**
Age		0.0197	0.0206	0.193	0.0160*
Sex		0.00298	−0.732	3.71	0.190
Height		−0.00343	−0.0842	0.129	0.684
Body weight		−0.00283	−0.0529	0.0952	0.576
Body mass index		−0.00324	−0.206	0.335	0.641
Heart rate at the examination		0.0121	−0.287	−0.00234	0.0475*
Systolic blood pressure		<0.0001	−0.0501	0.00580	0.891
Diastolic blood pressure		0.00202	−0.112	0.530	0.482
Clinical diagnosis	ECG abnormalities	0.0334	−6.01	−1.32	0.00242*
	Ischemic heart disease	0.0284	1.16	6.31	0.00483*
	Cardiomyopathy	−0.000728	−2.81	7.66	0.365
	Other	−0.00410	−2.04	2.12	0.973
Regular drug	α-blocker	0.000979	−1.48	5.37	0.267
	β-blocker	0.0250	0.787	5.01	0.00763*
	Ca-antagonist	0.00674	−0.372	4.01	0.105
	Diuretics	0.00726	−0.448	5.53	0.0969
	ARB/ACE-I	0.00798	−0.289	4.26	0.0869
Pre-study β-blocker	Bisoprolol fumarate	−0.00373	−1.80	2.48	0.756
	Metoprolol	0.00133	−4.40	1.15	0.251
	Landiolol	0.0227	0.654	4.80	0.0105*

*Statistically significant

CI, confidence interval; ARB, angiotensin II receptor blocker; ACE-I, angiotensin-converting enzyme inhibitor; ECG, electrocardiogram

**Table 5:** Factors associated with ΔEF analyzed by ordinary least squares regression models using the least squares method.

**Variable**	**Coefficient**	**Standard Error**	***t*-value**	**VIF**	***p*-value**
Age	0.0747	0.0440	1.71	3.54	0.102
Landiolol	1.96	1.03	1.90	2.34	0.0590
β-blocker (taken regularly)	2.68	1.12	2.42	1.81	0.016*
ARB/ACE-I (taken regularly)	1.00	1.13	0.89	1.38	0.377
Diuretics (taken regularly)	1.45	1.49	0.970	1.11	0.333
Ischemic heart disease	1.44	1.39	1.04	1.48	0.302
ECG abnormalities	−3.11	1.25	−2.48	1.51	0.014*

*Statistically significant

VIF, variance inflation factor; ARB, angiotensin II receptor blocker; ACE-I, angiotensin-converting enzyme inhibitor; ECG, electrocardiogram.

**Table 6:** Effects of the regular intake of β-blockers on cardiac function.

**-**	**Group**		***p*-value**
	**Taking β-Blockers Regularly**	**Not Taking β-Blockers Regularly**	
N	96	150	–
ESV (CT)	69.6	63.4	0.0379*
EDV (CT)	140	127	0.00470*
SV (CT)	70.3	64.1	0.0130*
DS (US)	32.2	30.5	0.0709
DD (US)	48.3	47.2	0.105

*Statistically significant.

CT, computed tomography; US, ultrasound; ESV, end-systolic volume; EDV, end-diastolic volume; SV, stroke volume; DS, end-systolic dimension; DD, end-diastolic dimension.

## Data Availability

The datasets generated during and analysed during the current study are available from the corresponding author [R.K.], on reasonable request.

## LIST OF ABBREVIATIONS

CCTA: Coronary Computed Tomography Angiography
HR: Heart Rate
ARB: Angiotensin II Receptor Blocker
Bpm: Beats Per Minute
CABG: Coronary Artery Bypass Grafting

## References

[1] Raff G.L., Gallagher M.J., O’Neill W.W., Goldstein J.A. (2005). Diagnostic accuracy of noninvasive coronary angiography using 64-slice spiral computed tomography.. J. Am. Coll. Cardiol..

[2] Rubinshtein R., Halon D.A., Gaspar T. (2007). Usefulness of 64-slice cardiac computed tomographic angiography for diagnosing acute coronary syndromes and predicting clinical outcome in emergency department patients with chest pain of uncertain origin.. Circulation.

[3] Levin D.C., Parker L., Halpern E.J., Rao V.M., Coronary C.T. (2019). Coronary CT angiography: Reversal of earlier utilization trends.. J. Am. Coll. Radiol..

[4] Taron J., Foldyna B., Eslami P., Hoffmann U., Nikolaou K., Bamberg F. (2019). Cardiac computed tomography - more than coronary arteries? A clinical update.. Röfo Fortschr. Geb. Röntgenstr. Neuen Bildgeb. Verfahr..

[5] Pannu H.K., Alvarez W., Fishman E.K. (2006). β-blockers for cardiac CT: A primer for the radiologist.. AJR Am. J. Roentgenol..

[6] Sabarudin A., Sun Z. (2013). Beta-blocker administration protocol for prospectively ECG-triggered coronary CT angiography.. World J. Cardiol..

[7] Abbara S., Blanke P., Maroules C.D. (2016). SCCT guidelines for the performance and acquisition of coronary computed tomographic angiography: A report of the society of Cardiovascular Computed Tomography Guidelines Committee: Endorsed by the North American Society for Cardiovascular Imaging (NASCI).. J. Cardiovasc. Comput. Tomogr..

[8] Leschka S., Wildermuth S., Boehm T. (2006). Noninvasive coronary angiography with 64-section CT: Effect of average heart rate and heart rate variability on image quality.. Radiology.

[9] Yancy C.W., Jessup M., Bozkurt B. (2017). 2017 ACC/AHA/HFSA focused update of the 2013 ACCF/AHA guideline for the management of heart failure: A report of the american college of cardiology/American heart association task force on clinical practice guidelines and the heart failure society of America.. Circulation.

[10] Nayler W.G., Chipperfield D., Lowe T.E. (1969). The negative inotropic effect of adrenergic betareceptor blocking drugs on human heart muscle.. Cardiovasc. Res..

[11] Bland J.M., Altman D.G. (1986). Statistical methods for assessing agreement between two methods of clinical measurement.. Lancet.

[12] Curtis J.P., Sokol S.I., Wang Y. (2003). The association of left ventricular ejection fraction, mortality, and cause of death in stable outpatients with heart failure.. J. Am. Coll. Cardiol..

[13] Smith G.L., Masoudi F.A., Vaccarino V., Radford M.J., Krumholz H.M. (2003). Outcomes in heart failure patients with preserved ejection fraction: Mortality, readmission, and functional decline.. J. Am. Coll. Cardiol..

[14] Vasan R.S., Larson M.G., Benjamin E.J., Evans J.C., Reiss C.K., Levy D. (1999). Congestive heart failure in subjects with normal versus reduced left ventricular ejection fraction: Prevalence and mortality in a population-based cohort.. J. Am. Coll. Cardiol..

[15] Folland E.D., Parisi A.F., Moynihan P.F., Jones D.R., Feldman C.L., Tow D.E. (1979). Assessment of left ventricular ejection fraction and volumes by real-time, two-dimensional echocardiography. A comparison of cineangiographic and radionuclide techniques.. Circulation.

[16] Nagueh S.F., Appleton C.P., Gillebert T.C. (2009). Recommendations for the evaluation of left ventricular diastolic function by echocardiography.. J. Am. Soc. Echocardiogr..

[17] Schiller N.B., Shah P.M., Crawford M. (1989). Recommendations for quantitation of the left ventricle by two-dimensional echocardiography. American Society of Echocardiography Committee on Standards, Subcommittee on Quantitation of Two-Dimensional Echocardiograms.. J. Am. Soc. Echocardiogr..

[18] Butler J., Shapiro M.D., Jassal D.S. (2007). Comparison of multidetector computed tomography and two-dimensional transthoracic echocardiography for left ventricular assessment in patients with heart failure.. Am. J. Cardiol..

[19] de Graaf F.R., Schuijf J.D., van Velzen J.E. (2010). Assessment of global left ventricular function and volumes with 320-row multidetector computed tomography: A comparison with 2D-echocardiography.. J. Nucl. Cardiol..

[20] Lim S.J., Choo K.S., Park Y.H. (2011). Assessment of left ventricular function and volume in patients undergoing 128-slice coronary CT angiography with ECG-based maximum tube current modulation: A comparison with echocardiography.. Korean J. Radiol..

[21] Schlosser T., Mohrs O.K., Magedanz A., Voigtländer T., Schmermund A., Barkhausen J. (2007). Assessment of left ventricular function and mass in patients undergoing computed tomography (CT) coronary angiography using 64-detector-row CT: Comparison to magnetic resonance imaging.. Acta Radiol..

[22] Collins J.E., Wali N., Sealy I.M. (2015). High-throughput and quantitative genome-wide messenger RNA sequencing for molecular phenotyping.. BMC Genomics.

[23] Wu Y.W., Tadamura E., Yamamuro M. (2008). Estimation of global and regional cardiac function using 64-slice computed tomography: A comparison study with echocardiography, gated-SPECT and cardiovascular magnetic resonance.. Int. J. Cardiol..

